# The herpes simplex virus host shutoff (vhs) RNase limits accumulation of double stranded RNA in infected cells: Evidence for accelerated decay of duplex RNA

**DOI:** 10.1371/journal.ppat.1008111

**Published:** 2019-10-18

**Authors:** Bianca Dauber, Holly A. Saffran, James R. Smiley

**Affiliations:** Department of Medical Microbiology and Immunology, University of Alberta, Edmonton, Alberta, Canada; Wistar Institute, UNITED STATES

## Abstract

The herpes simplex virus virion host shutoff (vhs) RNase destabilizes cellular and viral mRNAs and blunts host innate antiviral responses. Previous work demonstrated that cells infected with vhs mutants display enhanced activation of the host double-stranded RNA (dsRNA)-activated protein kinase R (PKR), implying that vhs limits dsRNA accumulation in infected cells. Confirming this hypothesis, we show that partially complementary transcripts of the UL23/UL24 and UL30/31 regions of the viral genome increase in abundance when vhs is inactivated, giving rise to greatly increased levels of intracellular dsRNA formed by annealing of the overlapping portions of these RNAs. Thus, vhs limits accumulation of dsRNA at least in part by reducing the levels of complementary viral transcripts. We then asked if vhs also destabilizes dsRNA after its initial formation. Here, we used a reporter system employing two mCherry expression plasmids bearing complementary 3’ UTRs to produce defined dsRNA species in uninfected cells. The dsRNAs are unstable, but are markedly stabilized by co-expressing the HSV dsRNA-binding protein US11. Strikingly, vhs delivered by super-infecting HSV virions accelerates the decay of these pre-formed dsRNAs in both the presence and absence of US11, a novel and unanticipated activity of vhs. Vhs binds the host RNA helicase eIF4A, and we find that vhs-induced dsRNA decay is attenuated by the eIF4A inhibitor hippuristanol, providing evidence that eIF4A participates in the process. Our results show that a herpesvirus host shutoff RNase destabilizes dsRNA in addition to targeting partially complementary viral mRNAs, raising the possibility that the mRNA destabilizing proteins of other viral pathogens dampen the host response to dsRNA through similar mechanisms.

## Author summary

Essentially all viruses produce double-stranded RNA (dsRNA) during infection. Host organisms therefore deploy a variety of dsRNA receptors to trigger innate antiviral defenses. Not surprisingly, viruses in turn produce an array of antagonists to block this host response. The best characterized of the viral antagonists function by binding to and masking dsRNA and/or blocking downstream signaling events. Other less studied viral antagonists appear to function by reducing the levels of dsRNA in infected cells, but exactly how they do so remains unknown. Here we show that one such viral antagonist, the herpes simplex virus vhs ribonuclease, reduces dsRNA levels in two distinct ways. First, as previously suggested, it dampens the accumulation of partially complementary viral mRNAs, reducing the potential for generating dsRNA. Second, it helps remove dsRNA after its formation, a novel and surprising activity of a protein best known for its activity on single-stranded mRNA. Many other viral pathogens produce proteins that target mRNAs for rapid destruction, and it will be important to determine if these also limit host dsRNA responses in similar ways.

## Introduction

Many if not all viruses produce double-stranded RNA (dsRNA) as an integral part of their life cycles: RNA viruses must generate complementary RNA species in order to replicate their genome, while DNA viruses produce complementary transcripts from overlapping diverging and converging transcription units. Host cells consequently deploy a variety of pattern recognition (PRRs) receptors to detect dsRNA and trigger innate antiviral responses, including the type I interferon system [1]. Not surprisingly, viruses in turn produce an array of antagonists to dampen the accumulation or recognition of dsRNA and/or interfere with downstream signaling events [2].

The host dsRNA-activated protein kinase R (PKR) is a key element of host RNA-based innate antiviral defenses [3, 4]. Following activation by binding to dsRNA or other activating ligands, PKR phosphorylates translation initiation factor eIF2α on serine 51, leading to a potent global blockade of translational initiation, severely impairing virus replication. Essentially every mammalian virus therefore encodes one or more PKR antagonists, which are required for efficient virus replication in cultured cells and virulence in the intact animal host [3].

Herpes simplex virus (HSV) is a large nuclear DNA virus and important human pathogen that encodes numerous immunomodulatory proteins [5], including three known PKR antagonists: the dsRNA binding protein US11, ICP34.5, and the virion host shutoff RNase vhs. US11 binds dsRNA and PKR and blocks PKR activation [6–8]. It also binds and inactivates PACT [9], a protein activator of PKR that acts in a dsRNA -independent fashion, and blocks the activity of several other PRRs that are activated by dsRNA, including OAS [10] and RIG-I [11]. ICP34.5 serves as an adaptor that recruits protein phosphatase 1α to dephosphorylate eIF2α,[12]. In contrast to the well described mechanisms of US11 and ICP34.5 action, relatively little is known about how vhs antagonizes PKR [13, 14].

Vhs is a FEN-1 family endoribonuclease that serves to shut off host protein synthesis [15, 16], a strategy shared with other viruses that encode mRNA destabilizing proteins, including pathogens such as poxviruses, influenza A virus, γ-herpesviruses and coronaviruses (reviewed in [15, 17–20]). Vhs is packaged into the virion tegument (the space between the viral capsid and envelope) and delivered into host cells immediately upon infection. Vhs globally destabilizes host mRNAs, thereby suppressing host antiviral responses that require new protein synthesis. It directly binds to host translation initiation factors eIF4A (an RNA helicase) and eIF4H (an eIF4A co-factor), components of the cap-binding complex eIF4F [21–24]. The interaction with eIF4F is thought to target vhs to the capped 5’ ends of mRNA [21, 25], accounting for why vhs targets mRNAs and spares other cytoplasmic RNA species [18, 26, 27].

Vhs also destabilizes viral mRNAs [27], facilitating the successive transitions between the immediate-early (IE), early (E), and late (L) phases of viral gene expression by strongly coupling changes in the transcription rates of viral genes to rapidly altered mRNA levels [26–28]. Thus, when vhs is inactivated, most cellular mRNAs and viral IE and E mRNAs persist until late times post-infection, rather than sharply declining in abundance as during infection with wild-type HSV. In contrast, vhs has relatively little effect on the levels of viral L transcripts, at least in part because vhs RNase activity is downregulated late during infection through interactions between vhs and additional viral proteins including VP16, UL47, and VP22 [29–32].

In addition to shutting off host protein synthesis and downregulating viral IE and E gene expression at late times post-infection, vhs blunts host innate antiviral and stress responses [13, 14, 33–37], and stimulates the expression of viral L genes [36]. Although deleting vhs has little impact in some cell lines such as Vero cells, viral replication is reduced ca. 50-fold in other cell lines such as HeLa cells [36]. In such restrictive cells, vhs null mutants trigger robust PKR activation [13, 38] and formation of stress granules (SG, [36, 37, 39]); in addition, translation of viral true late (L2) mRNAs is severely impaired at the level of initiation [36, 40]. The ability of vhs to suppress PKR and cellular stress responses and stimulate viral protein synthesis is shared by the mRNA destabilizing proteins of other viruses, illustrating important commonalities between the regulatory strategies of diverse viral pathogens (see for example [19, 20, 41–44]).

Based on the known properties of vhs, at least three distinct mechanisms of PKR suppression can be envisioned, which are not mutually exclusive. First, vhs-induced disruption of SGs could interfere with the recently described SG-dependent feed-forward pathway of PKR activation [45, 46], dampening PKR activity. Strongly supporting this idea, Burgess and Mohr recently showed that PKR activation by vhs-null HSV is reduced when SG formation is blocked [47]. Second, vhs is required for efficient expression of US11 (an L2 protein) in restrictive cell lines [36], perhaps accounting for some or all of its inhibitory effects on PKR in such cells. Third, vhs might somehow limit the accumulation of dsRNA in infected cells, perhaps through its nuclease activity [13, 35]. This hypothesis is plausible and has been broadly accepted in the field, but it has not been directly tested. Consistent with the proposal, Burgess and Mohr [47] found that cells infected with vhs-null HSV display enhanced reactivity with a dsRNA-specific monoclonal antibody (J2,[48]) in an immunofluorescence assay. However, as we describe below, US11 can shield dsRNA from the J2 mAb in such assays, potentially complicating the interpretation of the results.

Here we delineate the roles of vhs and US11 in PKR suppression during infection of permissive and restrictive human cells, and provide direct evidence that vhs limits the accumulation of dsRNA, including species arising from complementary viral transcripts. We also present evidence that vhs destabilizes preformed dsRNA and dsRNA-US11 complexes, a novel and perhaps unexpected activity of vhs. These findings significantly advance our understanding of how a viral shutoff ribonuclease dampens host RNA-based innate immune responses, findings that are likely relevant to other viral pathogens that encode RNA destabilizing proteins.

## Results

### Vhs reduces the levels of dsRNA in infected cells

**Fig 1:**
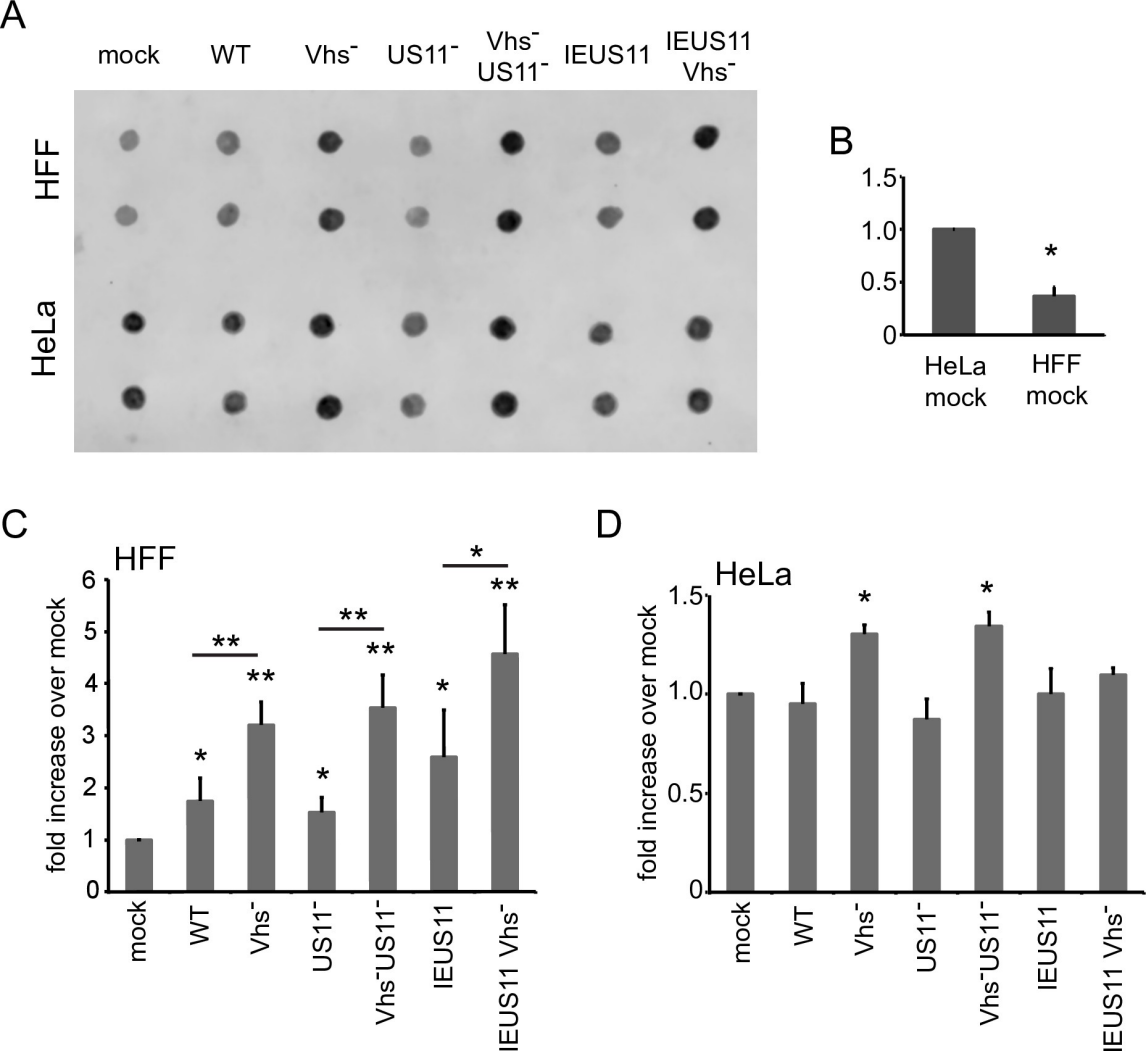
Accumulation of dsRNA in cells infected with vhs-deficient viruses. **A)** HFF and HeLa cells were infected for 12 hours with HSV-1 (KOS37) WT, Vhs^-^, US11^-^, Vhs^-^US11^-^, IEUS11 and IEUS11 Vhs^-^ at an MOI of 10. Total RNA was extracted and 1μg in 1μl was spotted onto a nylon membrane, UV-crosslinked and processed like a Western blot. DsRNA was detected by staining with the dsRNA-specific antibody J2. **B)** Quantification of dsRNA in mock-infected HeLa and HFF cells from 5 independent experiments, * p< 0.0005; **C)** Quantification of dsRNA signal in HSV- and mock-infected HFF cells from 6 independent experiments, * p<0.05, ** p<0.0005; **D)** Quantification of dsRNA signal in HSV- and mock-infected HeLa cells from 3 independent experiments, * p<0.05.

As reviewed in the Introduction, vhs has been proposed to limit PKR activation at least in part by reducing the amount of dsRNA in infected cells; however, the levels of dsRNA during infection with wild-type and vhs-null HSV have not been directly measured. We therefore assessed dsRNA levels using a dot-blot immuno-assay for RNA recognized by the dsRNA-specific J2 monoclonal antibody [48], which binds dsRNAs greater than 40 nt in length (Fig 1). HeLa cells and telomerase-immortalized human foreskin fibroblast (HFF) cells were infected with wild-type HSV-1 strain KOS37 and KOS37-derived mutants that lack functional vhs (Vhs^-^), US11 (US11^-^), or both proteins (Vhs^-^ US11^-^). In addition, we assessed vhs+ and vhs- KOS37 derivatives that over-express US11 from the viral immediate-early ICP47 promoter (IEUS11 and IEUS11 Vhs^-^). Total cellular RNA extracted 12 hours post-infection was spotted onto positively charged Nylon membranes (1 μg in 1 μl), UV-cross-linked, then scored for dsRNA using the J2 mAb (Fig 1). Control experiments revealed that the signal obtained in this assay was not affected by treating the purified RNA with a combination of the single-strand-specific ribonucleases RNase A/T1, but was eliminated by RNase III, which degrades dsRNA, confirming it arises from dsRNA (S1 Fig).

In HFF cells, the vhs+ viruses (WT, US11^-^, and IEUS11) induced a small but significant increase in dsRNA levels compared to mock-infected cells, while the corresponding vhs-deficient derivatives induced a larger increase (Fig 1C). Uninfected HeLa cells consistently yielded higher levels of dsRNA than HFF (Fig 1B), indicating that endogenous dsRNAs are more abundant in this cell line. While the nature of the endogenous dsRNA was not determined, it is likely derived in part from transcripts bearing inverted Alu SINE elements [49, 50]. The impact of HSV and vhs on total dsRNA levels was less clear-cut in HeLa cells than in HFF cells, likely due to the higher levels of endogenous dsRNA, but in this case as well the vhs- viruses provoked a small but significant increase in dsRNA levels compared to the WT virus (Fig 1D). Overall, these data indicate that vhs acts directly or indirectly to lower the levels of dsRNA in both cell lines. Although these experiments do not address the origin of the dsRNAs that accumulate in the absence of vhs, we demonstrate below that at least some arise from the viral genome, a finding that is consistent with the observation that PKR activation by Vhs- virus is blocked by inhibiting viral DNA replication and hence the accumulation of viral L transcripts (38).

### Vhs and US11 make independent contributions to PKR suppression during infection

**Fig 2:**
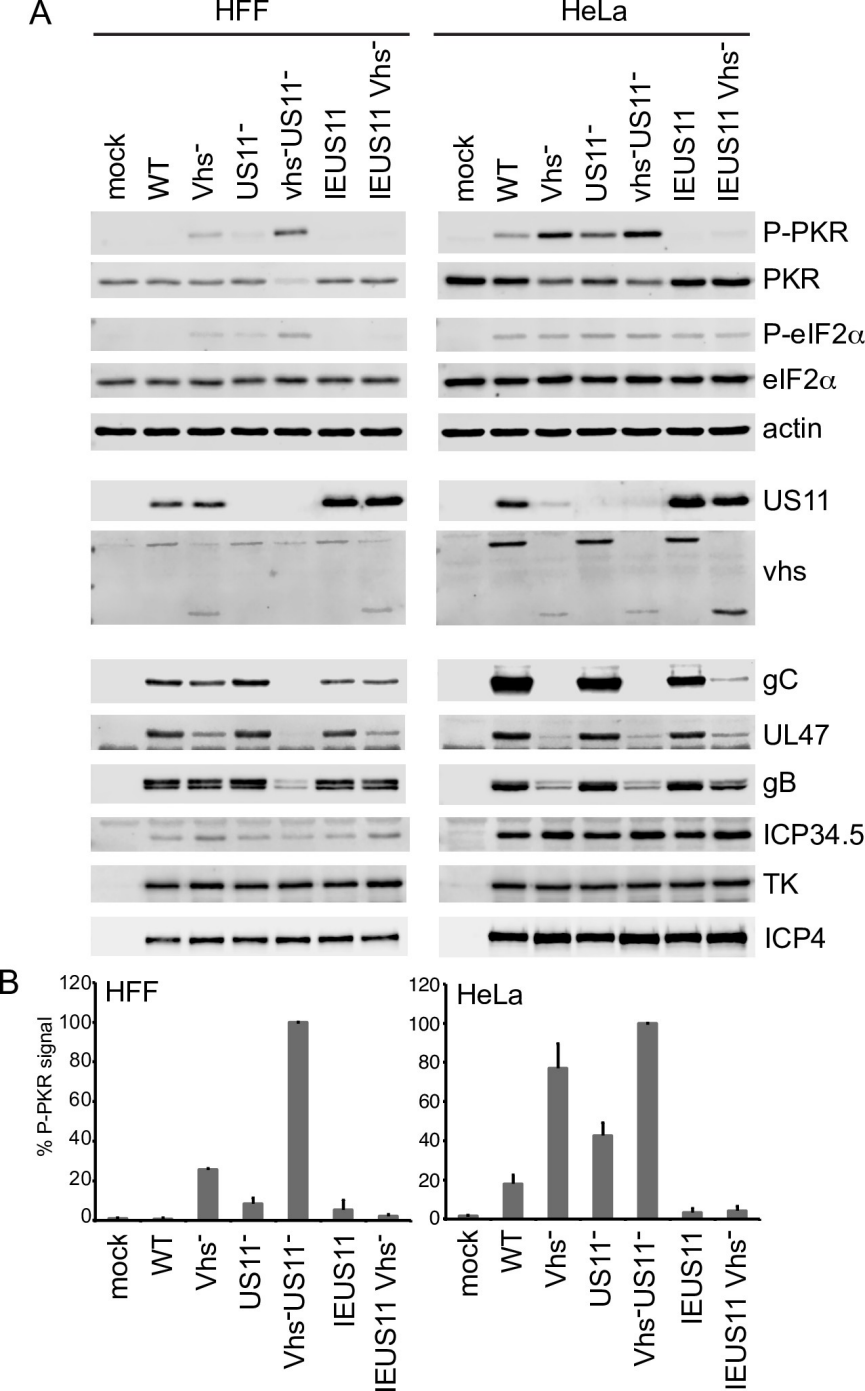
Vhs and US11 make independent and complementary contributions to PKR suppression during HSV infection. HFF and HeLa cells were infected for 12 hours with HSV-1 (KOS37) WT, Vhs^-^, US11^-^, Vhs^-^US11^-^, IEUS11 and IEUS11 Vhs^-^ at an MOI of 10. **A)** Whole-cell lysates were analyzed by Western blot with the indicated antibodies. Note that the Vhs antibody used cross-reacts with a co-migrating cellular protein present in HFF cells. **B)** Quantification of the P-PKR signal in HFF and HeLa cells. Data are from 4 independent experiments. The P-PKR signal in cells infected with Vhs^-^US11^-^ was set at 100%.

Vhs is required for efficient accumulation of US11 and other HSV true L proteins in some cell lines but not others [36]. This difference allowed us to uncouple the effects of vhs on US11 levels from its impact on PKR activation. In HFF cells, where US11 levels are largely independent of vhs [51], inactivating vhs provoked elevated levels of PKR activation and eIF2α phosphorylation compared to WT virus, while inactivating US11 had a smaller but still significant effect (Fig 2). Cells infected with the Vhs^-^US11^-^ double mutant displayed higher levels of PKR activation and eIF2α phosphorylation than those infected with either singly mutated virus, accompanied by severely reduced levels of the viral L2 proteins gC, UL47 and the internally deleted form of vhs produced by ΔSma [52], and moderately reduced levels of gB (L1). Little effect was observed on expression of ICP34.5 (E), tk (E), and ICP4 (IE). These results indicate that vhs and US11 make independent contributions to PKR suppression in these cells, and that when both are missing PKR is hyperactivated and expression of viral L genes is impaired. A slightly different picture emerged in HeLa cells, where US11 expression depends heavily on vhs [36]. Here as well, deleting vhs or US11 provoked elevated levels of PKR activation, but deleting US11 in the vhs- background had relatively little effect beyond that achieved by inactivating vhs alone, likely because Vhs^-^ is already largely US11-deficient in these cells. Significantly, in both cell types, overexpressing US11 from the IE ICP47 promoter in the vhs- background (IEUS11/Vhs^-^) effectively suppressed PKR activity, showing that US11 can overcome the effect of inactivating vhs if it is expressed to sufficiently high levels. Overall, these data indicate that vhs and US11 make independent and complementary contributions to PKR suppression when expressed at physiological levels during infection; that is, both proteins are required to fully suppress PKR activity.

### Vhs reduces accumulation of dsRNA derived from partially complementary viral transcripts

**Fig 3:**
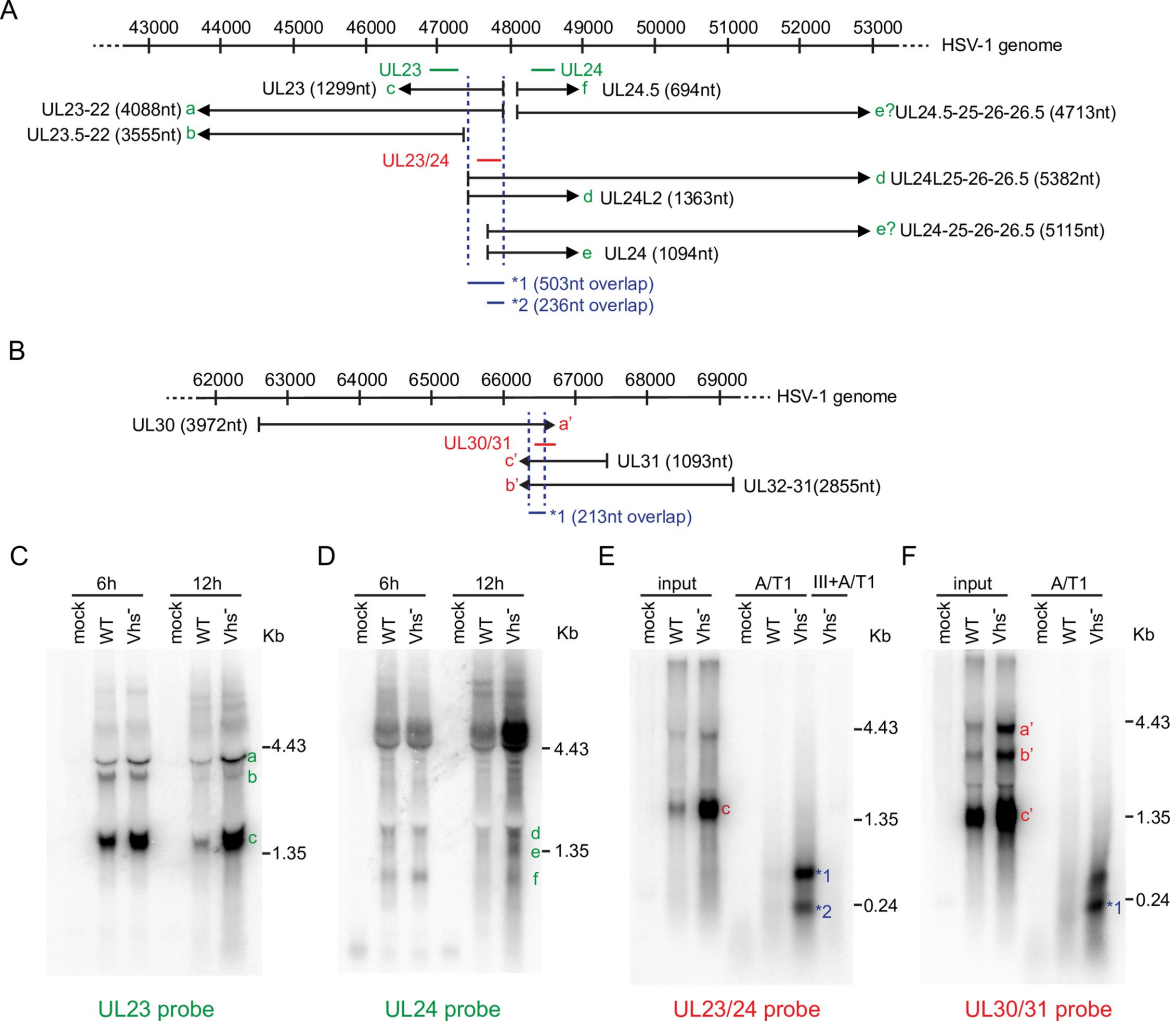
Effects of vhs on the levels of partially complementary viral transcripts and dsRNA levels. **A and B)** Diagram of the UL23/UL24 (A) and UL30/UL31 (B) regions of the HSV-1 genome, with the transcripts detected by Tombacz et al. 2017 and earlier studies depicted. The probes used for northern blots analysis are diagrammed. Coordinates are from the HSV-1 strain 17 reference genome (X14112). **C** and **D)** HeLa cells were infected with HSV-1 (KOS) WT and Vhs^-^ for 6 or 12 hours at an MOI of 10. Equal amounts of total RNA were analyzed by Northern blot with probes specific for either the UL23 (C) or UL24 (D) transcripts. Results shown are representative of 3 independent experiments. **E)** Detection of UL23/UL24 duplex RNA. HeLa cells were infected with HSV-1 (KOS) WT and Vhs^-^ for 12 hours at an MOI of 10. Total RNA was extracted and digested with RNaseA/T1 (high salt buffer) or RNaseIII followed by RNaseA/T1. Undigested input (1/10) and digested samples were analyzed by Northern blot with a probe specific for the predicted overlap between the UL23 and UL24 transcripts. **F)** Detection of UL30/UL31 duplexRNA. The experiment was performed as in E) but with a probe specific for the predicted overlap between UL30 and UL31 transcripts. Results shown in E) and F) are representative of 4 independent experiments each.

Classical studies showed that HSV and other DNA viruses produce complementary transcripts with the potential to form dsRNA [53–55], and provided detailed descriptions of several such sets of complementary HSV-1 RNAs. For example, the UL23 (thymidine kinase) and UL24 open reading frames are arranged in a partially overlapping head-to-head fashion [56], with two separately promoted UL24 transcripts overlapping the 5’ region of UL23 mRNA [57–61]. These UL23 and UL24 transcripts have the potential to anneal to produce dsRNA regions ca. 240 and 500 nt in length. More recent next generation long-read RNA sequence analysis has confirmed these findings and provided a more detailed picture of the multiple UL23 and UL24-related transcripts arising from this region of the viral genome ([61], summarized in Fig 3A). Similarly, the converging UL30 (DNA polymerase) and UL31 and UL32-31 transcripts [56, 61] could potentially form dsRNA in the ca. 210 nt region of overlap (Fig 3B).

We evaluated the impact of vhs on the accumulation of the UL23 and UL24-related transcripts by analyzing RNA harvested 6 and 12 hours postinfection by northern blot, using UL23 and UL24-specific probes (depicted in green in Fig 3A). Consistent with previous work [26], deleting vhs prevented the decline of UL23 mRNA that occurs at late times during infection with WT HSV (Fig 3C, b and c). Similarly, the vhs mutant displayed elevated levels of most or all of the UL24-related transcripts at 12 hours post-infection, an effect that was especially evident for the >4.4 kb species that extend into the downstream UL25 and UL26 regions (Fig 3D). Thus, vhs dampens the accumulation of the partially complementary UL23 and UL24 RNAs at late times post-infection, limiting the potential for formation of dsRNA in the region of overlap. To determine if such dsRNA indeed accumulates in the absence of vhs, we treated the purified RNA with the single strand-specific RNases A and T1, then analyzed the resistant material by northern blot using a probe derived from the region of complementarity (depicted in red in Fig 3A). Although little dsRNA was detected in the RNA from cells infected with WT HSV-1, protected species of the predicted sizes were readily detected with RNA from cells infected with the vhs-deficient virus (species 1 and 2, Fig 3E). These were eliminated by treating the samples with the dsRNA-specific nuclease RNase III, confirming that they represent dsRNA. Similar results were obtained with converging transcripts arising from the UL30 and UL31 genes: the vhs mutant displayed elevated levels of the transcripts relative to WT virus at 12 hours post-infection, and the vhs-deficient RNA sample gave rise to two dsRNA species that hybridize to a probe for the region of overlap (Fig 3F) following digestion with RNAs A/T1. One of these (band 1) is approximately the length of the known region of complementarity (ca. 210 nt.); the origin of the somewhat longer dsRNA species has not been determined.

**Fig 4:**
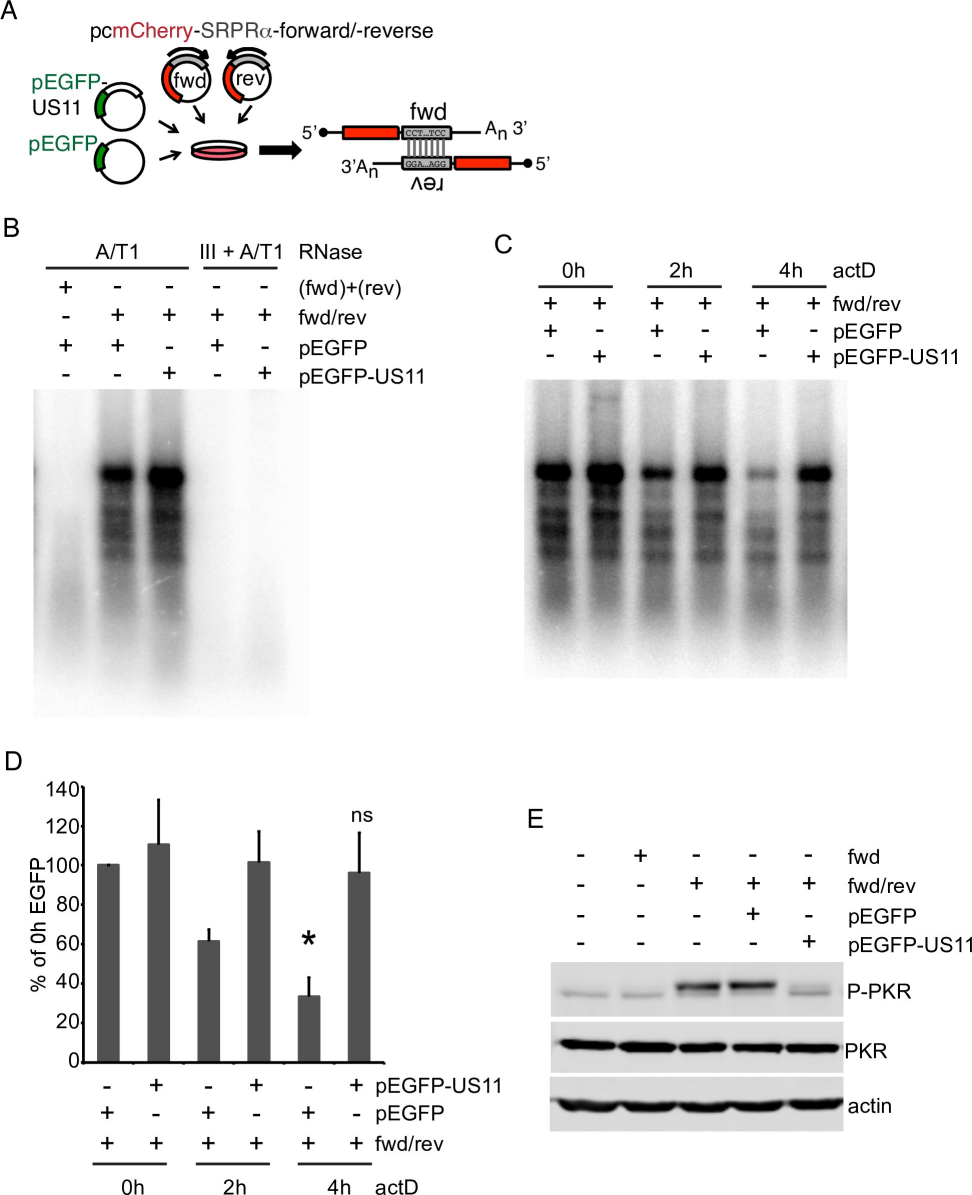
Effects of US11 on the stability and immune signaling activity of duplex RNA formed by annealing of partially complementary reporter mRNAs. **A**) Schematic of dsRNA generation. MCherry expression plasmids with a fragment of the SRPRα ORF (canine signal recognition particle receptor subunit alpha) inserted in the 3’UTR in the forward (fwd) or reverse (rev) orientation. Co-transfection of both plasmids results in formation of dsRNAs with 5’ and 3’ overhangs. **B**) HeLa cells were transfected with fwd and rev together (fwd/rev) along with either pEGFP or pEGFP-US11 for 20 hours. As a control, cells in separate dishes were transfected with either fwd/pEGFP or rev/pEGFP and combined during Trizol lysis (fwd)+(rev). Total RNA was extracted, digested by DNaseI and RNaseA/T1 (high salt conditions) or DNaseI, RNaseIII and RNaseA/T1 and analyzed by Northern blot with a probe specific for the inserted SRPRα fragment. **C)** Cotransfection of fwd/rev and pEGFP or pEGFP-US11, respectively. Transfected cells were treated with actinomycin D for 0, 2 or 4 hours prior to RNA extraction and processed as in (B). **D**) Quantification of the full-length dsRNA SRPRα signal from 3 independent experiments. P-value for pEGFP/4h vs. 0h is 0.010, 4h/pEGFP-US11 is ns/nonsignificant. **E)** HeLa cells were transfected with fwd or fwd/rev and pcDNA, pEGFP or pEGFP-US11, respectively. After 20 hours cells were treated with actinomycinD for 4 hours. Whole-cell lysates were analyzed by Western blot with the indicated antibodies. Results shown are representative of 3 independent experiments.

These results indicate that vhs reduces the levels of the partially complementary transcripts from the UL23/UL24 and UL30/U31 regions of the viral genome, and suppresses the accumulation dsRNA formed by annealing of the regions of complementarity. However, a caveat of the latter conclusion is that these experiments do not exclude the possibility that significant levels of RNA annealing occur during the RNA extraction process. Direct evidence that this is not the case under our experimental conditions is presented below (Fig 4).

### US11 stabilizes dsRNA

Endogenous dsRNAs are rapidly processed and degraded in uninfected cells, limiting activation of PKR and other cellular dsRNA-specific PRRs (see for example [49, 50, 62–64]). Given that US11 suppresses PKR activation at least in part by blocking dsRNA recognition, it was of great interest to explore the effects of US11 and vhs on the stability of dsRNA. To this end, we developed a plasmid-based system to produce defined dsRNA species in uninfected cells (Fig 4A). The system employs two mCherry expression plasmids that bear a 670 nt sequence inserted into the 3’ UTR, in either the forward (fwd) or reverse (rev) orientation. Thus, cells cotransfected with both plasmids should produce a pair of partially complementary RNAs with the potential of forming a 670 nt region of dsRNA via intermolecular hybridization (Fig 4A). To test the system, HeLa cells were co-transfected with both plasmids (fwd/rev) along with plasmids encoding either eGFP or an eGFP-US11 fusion protein, and RNA extracted 24 hours later was scored for the presence of dsRNA by northern blot analysis after digestion with RNase A/T1 (Fig 4B). As a control, lysates of cells that had been singly transfected with the fwd or rev constructs were mixed together, and the RNA present in the mixture was purified and analyzed in the same way (Fig 4B, (fwd) + (rev)). RNA from co-transfected cells gave rise to a strong signal of the predicted length, along with several discrete smaller species. All of these protected RNAs were eliminated by RNase III digest, confirming their double-stranded nature. In contrast, no dsRNA signal was obtained with the RNA extracted from the mixture of singly transfected cells, demonstrating that the complementary RNAs do not detectably anneal during the RNA purification procedure. The origin of the less than full length dsRNA species remains unclear; one possibility is that they are derived from the full-length duplex region by nuclease action, perhaps following A->I editing of the duplex region by double-stranded RNA-specific adenosine deaminases such as ADAR1 (see discussion). We consistently observed a stronger dsRNA signal when the eGFP-US11 expression plasmid was included in the transfection cocktail with the fwd and rev plasmids, raising the possibility that US11 stabilizes the dsRNA region. This hypothesis was confirmed in an actinomycin D chase experiment (Fig 4C). Here, cells were transfected as before, incubated for 20 hours, then actinomycin D was added to block new transcription of the reporter RNAs. The fate of the dsRNA region was then followed for 4 hours. In cells expressing eGFP, the dsRNA signal rapidly decayed over time, with loss of the intact signal being especially pronounced; however, this loss was greatly attenuated in cultures expressing the eGFP-US11 fusion protein (Fig 4C, quantified in Fig 4D). This result implies that US11 inhibits the cellular dsRNA degradation/processing machinery, and/or blocks its access to the duplex RNA.

Cells co-expressing the fwd and rev constructs displayed robust PKR activation which was blocked by the eGFP-US11 fusion (Fig 4E), while PKR was not activated in cells expressing only the fwd (or reverse, S2 Fig) construct. These results demonstrate that the duplex RNAs produced in our reporter system are competent to stimulate a dsRNA-based innate immune response, and that the eGFP-US11 fusion protein used in our experiments is functionally active.

Overall the results outlined in this section document that the predicted dsRNA species accumulate in the cotransfected cells, activating a strong PKR response. The duplex RNA is unstable, but US11 interferes with its degradation and/or processing, likely by binding the dsRNA and shielding it from the relevant cellular machinery in the same fashion that it masks dsRNA from PKR, blocking PKR activation.

### Vhs destabilizes dsRNA and reverses PKR activation

**Fig 5:**
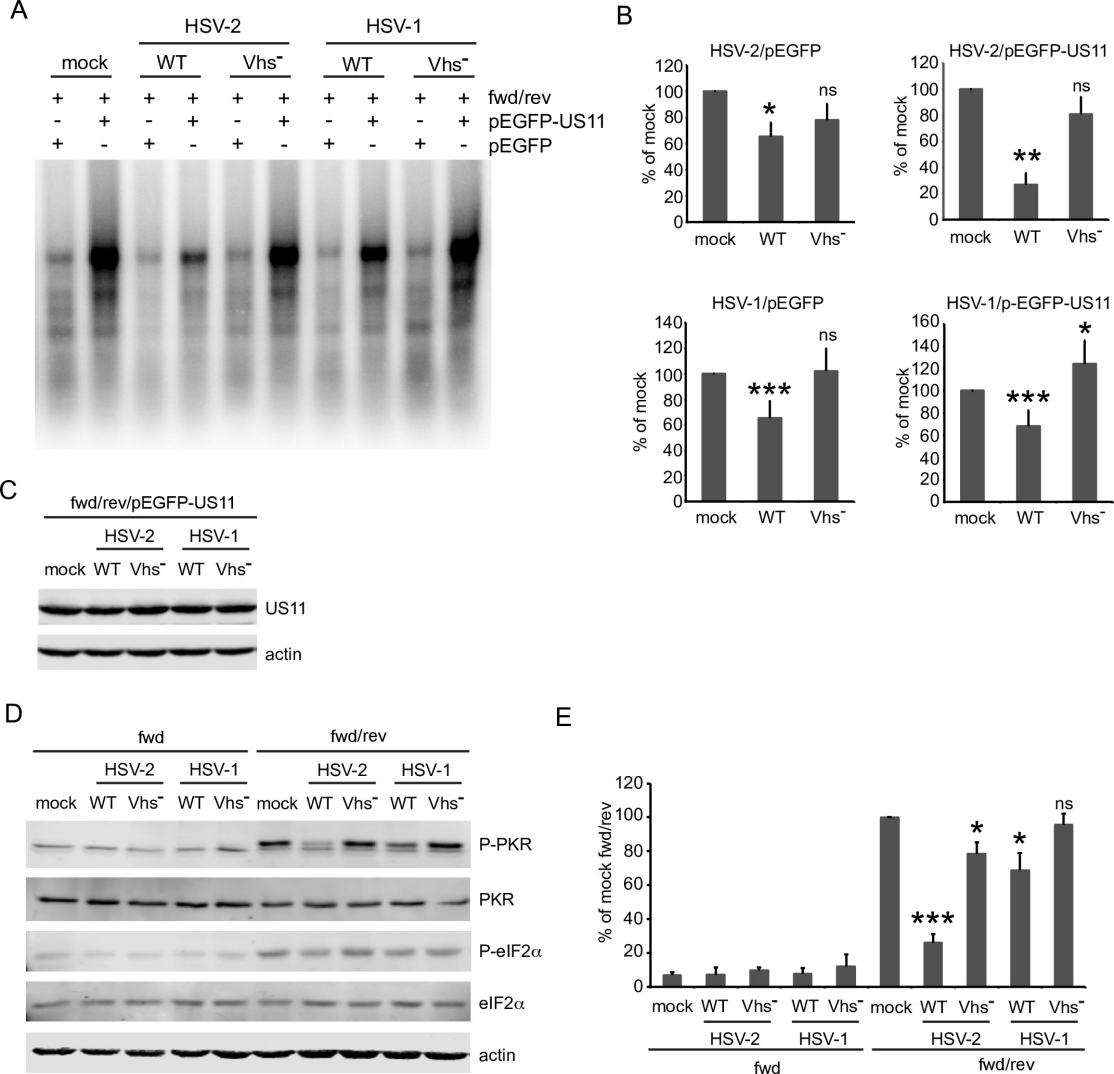
Virion-derived vhs accelerates decay of ectopically expressed dsRNA and concomitantly reverses PKR activation. **A**) HeLa cells were transfected with fwd/rev and either pEGFP or pEGFP-US11 for 20 hours. Cells were mock infected or infected with HSV-1 (KOS) and HSV-2 WT and Vhs^-^ at MOI 20 for 4 hours with continuous actD treatment. Total RNA was extracted, digested by DNaseI and RNaseA/T1 (high salt conditions) and analyzed by Northern blot with a probe specific for the inserted SRPRα fragment. **B**) Quantification of full-length SRPRα dsRNA from 3 (HSV-2) or 6 (HSV-1) independent experiments. P-values vs. mock: * p<0.05, ** p< 0.01, *** p<0.005, ns/nonsignificant; P-values for WT vs. Vhs^-^: ns (HSV-2/pEGFP), p<0.05 (HSV-2/pEGFPUS11), and p<0.005 (HSV-1); **C**) Cells were transfected with fwd/rev and pEGFP-US11 for 20 hours and infected as described in A). Whole cell lysates were analyzed by Western blot with the indicated antibodies. Results shown are representative of 3 independent experiments. **D**) Virion-derived vhs reverses PKR activation by preformed dsRNA. Cells were transfected with fwd/rev and pEGFP for 20 hours and infected as described in A). Whole cell lysates were analyzed by Western blot with the indicated antibodies. **E)** Quantification of P-PKR levels from 3 independent experiments. P-values vs. fwd/rev/mock: *p< 0.02, *** p<0.0002, HSV-2 WT vs. Vhs^-^ p<0.0001, HSV-1 WT vs. Vhs- p<0.02.

We next explored the impact of vhs on the stability of the dsRNA region in the presence and absence of US11. Cells were co-transfected with the fwd and rev plasmids as before and incubated 20 hours to allow the accumulation of dsRNA and the eGFP-U11 fusion protein. Actinomycin D was then added to halt transcription, and functional vhs was delivered by infecting the cells with wild type HSV-1 or HSV-2, with vhs- viruses serving as the negative control. The cells were then incubated a further 4 hours in the presence of actinomycin D, and RNA was scored for the duplex region as described above. Note that this experiment assesses the impact of vhs delivered by infecting HSV virions in the absence of viral gene expression, a classic assay for vhs activity. As observed previously, co-transfection of the eGFP-US11 expression plasmid greatly increased the levels of dsRNA remaining after the 4 hour actinomycin D chase in mock-infected cells. Wild-type HSV-1 and HSV-2 virions lowered the levels of dsRNA in both the presence and absence of US11 in a vhs-dependent fashion, with the effect being especially pronounced with HSV-2 in the presence of US11 (Fig 5A, results quantified in Fig 5B). The superinfecting virions had no detectable impact on the levels of the eGFP-US11 fusion protein over the course of the experiment, arguing against the possibility that reduced levels of US11 contribute to the effect (Fig 5C). These data indicate that vhs destabilizes dsRNA and dsRNA-US11 complexes. This effect appears to be functionally relevant, as the wild-type virus particles were able to partially reverse PKR activation induced by the cotransfected complementary RNAs, an effect that was vhs-dependent (Fig 5D, quantified in Fig 5E).

**Fig 6:**
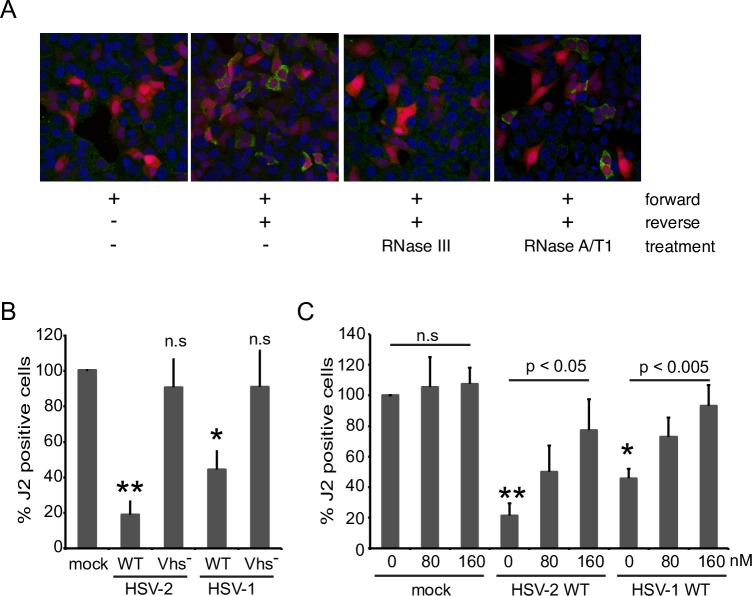
Reduction of preformed dsRNA by virion-derived vhs is sensitive to hippuristanol. **A)** HeLa cells were transfected with fwd or fwd/rev for 20 hours. Cells were then mock treated or treated with RNaseIII or RNaseA/T1 (high salt buffer) and analyzed by immunofluorescence with the dsRNA-specific antibody J2 (green). **B)** HeLa cells were transfected with fwd/rev for 20 hours and mock infected or infected with HSV-1 (KOS) and HSV-2 WT and Vhs^-^ at an MOI of 20 for 5 hours in the continuous presence of actinomycin D. After immunofluorescence staining with the J2 antibody the ratio of J2 positive cells was determined by analyzing more than 100 mCherry-positive cells per coverslip. The ratio of J2 positive to mCherry positive cells in the mock infected sample was set as 100%. The graph represents the average of 4 independent experiments. P-value vs. mock: * p<0.005, ** p< 0.0005; ns: nonsignificant; **C)** Hippuristanol (hip) antagonizes the dsRNA-destabilizing effect of vhs. The experiment was performed as described in B) with addition of vehicle control (0nM) or hip at 80 or 160nM during the course of infection. The graph represents the average of 4 independent experiments. P-values WT 0nM hip vs. mock 0nM hip: * p<0.005, ** p<0.0005; p-value 160nM hip vs. 0nM hip: p<0.05 (HSV-2), p<0.005 (HSV-1).

As an independent test of these findings, we examined the effects of vhs delivered by infecting HSV virions on the levels of intracellular dsRNA detected by the J2 monoclonal antibody (Fig 6). Control experiments showed that approximately 30–40% of cells co-transfected with the fwd and rev constructs display a dramatically stronger cytoplasmic J2 signal than cells transfected only with the fwd construct in an immunofluorescence assay (Fig 6A). This signal was resistant to RNase A/T1 and eliminated by RNase III, indicating that it arises from dsRNA. Interestingly, the J2 dsRNA signal was strongly suppressed by co-expressing the eGFP-US11 fusion protein but was unaffected by co-expressed eGFP (S3 Fig). Given that the eGFP-US11 fusion protein increases the amount of dsRNA detected by northern blotting (Fig 4), this result implies that US11 blocks dsRNA recognition by the J2 antibody. This finding therefore cautions against the use of the J2 immunofluorescence assay to assess dsRNA levels in HSV-infected cells, where high levels of US11 are produced at late time post-infection. Following accumulation of dsRNA, the co-transfected cells were super-infected with wild-type and vhs-deficient HSV-1 and HSV-2 in the presence of actinomycin D, then fixed and processed for immunofluorescence microscopy 5 hours later. HSV-1 and HSV-2 strongly reduced the proportion of J2-reactive cells in a vhs-dependent fashion, with HSV-2 displaying somewhat more robust activity (Fig 6B). Note that this assay was conducted in the absence of *de novo* viral gene expression and that HSV virions do not contain US11 protein [65], arguing against a confounding effect of US11 in these experiments.

Vhs has been characterized as an mRNA-specific endoribonuclease, and so its ability to reduce the stability of dsRNA was somewhat unexpected. As described in the Introduction, vhs directly binds the RNA helicase eIF4A, an interaction that has been proposed to help target vhs to the capped 5’ ends of mRNA. As a first step in characterizing cellular co-factors required for vhs-induced destabilization of dsRNA, we tested the impact of the eIF4A inhibitor hippuristanol on this activity. Strikingly, 160 nM hippuristanol significantly impaired the loss of J2-reactivity triggered by HSV-1 and HSV-2 (Fig 6C), suggesting eIF4A may play an important role in dsRNA destabilization.

## Discussion

We have reached three major conclusions based on the results presented in this communication. First, the HSV virion host shutoff RNase vhs limits the accumulation of dsRNA in infected cells, as long suspected on the basis of indirect evidence. Second, vhs likely does so both by reducing the levels of complementary viral transcripts (as previously suggested), and by destabilizing dsRNA after its formation–a novel and unanticipated activity of vhs. Third, US11 stabilizes dsRNA and vhs counteracts this effect, thereby providing the virus with another level of protection from dsRNA-based antiviral responses.

Our results show that elevated levels of dsRNA derived from the viral genome accumulate in infected cells when vhs is inactivated (Fig 3). These arise by annealing of complementary viral transcripts, forming relatively long (>200 nt) fully duplexed regions. Our study focused on the dsRNAs produced from two regions of the viral genome that encode well-characterized partially complementary mRNAs (UL23/24 and UL30/31), and further more detailed analyses will be required to fully define the set of viral dsRNAs that accumulate in the absence of vhs. These findings confirm earlier predictions that vhs limits accumulation of dsRNA [13, 35, 47], and can explain, at least in part, why vhs mutants display elevated PKR activity [13, 14, 47]. Combined with our finding that vhs-dependent PKR inhibition can be uncoupled from the effect of vhs on US11 protein levels (Fig 2), these results establish vhs as a *bona-fide* modulator of dsRNA-based innate antiviral responses.

How does vhs dampen accumulation of dsRNA? Several groups have suggested that it does so by limiting the accumulation of complementary viral transcripts [13, 35, 47]; consistent with this hypothesis, it has long been known that vhs destabilizes both host and viral mRNAs [26, 27], and that vhs mutants therefore display increased levels of many viral transcripts at late times post-infection relative to wild-type HSV [27, 35, 26]. Our data show that such over-expressed mRNAs include the partially complementary species from the UL23/UL24 and UL30/UL31 regions and that these transcripts actually form duplex structures, strongly supporting the proposal. It is possible that vhs additionally limits the potential for dsRNA formation by spatially segregating such complementary viral transcripts. For example, Pheasant et al. [66] have shown that vhs activity sequesters viral IE and E transcripts to the nucleus at late time post-infection, while L transcripts localize to the cytoplasm. This process could separate the E UL23 and UL30 mRNAs from their partially complementary potential partners, the L UL24 and UL31 mRNAs.

Our data also provide strong evidence that US11 and vhs modulate the stability of dsRNA after its formation (Figs 5 and 6). The RNase A/T1- resistant duplex RNA signal arising from our partially complementary reporter mRNAs rapidly decayed in uninfected cells when transcription was blocked, (Fig 4), indicating that the dsRNA is either degraded and/or processed to forms susceptible to attack by the single-strand specific RNases used in our assay. Further studies are required to distinguish between these possibilities and to identify the host factors involved. Potential candidates include dsRNA-specific adenosine deaminases such as ADAR1, which destabilize duplex RNA structures by A to I editing (reviewed in [67], the nucleases Tudor-SN and endonuclease V that can cleave A->I edited duplex regions [68, 69], dicer [62], and Staufen 1 [70], which mediates degradation of mRNAs bearing duplex regions in their 3’ UTR [63, 64].

US11 markedly stabilized the duplex region (Fig 5), suggesting that it shields the dsRNA from the relevant processing/degradation machinery in the same way that it blocks recognition by PKR and other PRRs that recognize dsRNA. The ability of US11 to stabilize dsRNA in our transfection assay is intriguing and somewhat counterintuitive, and it will be important to determine if US11 similarly stabilizes dsRNA arising from the viral genome during infection, and if the dsRNA binding proteins of other viruses have similar effects. Indeed, we found that over-expressing US11 in the IEUS11 Vhs- virus results in a significant increase in the total amount of dsRNA compared to Vhs- virus in HFF cells (Fig 1C, p = 0.023), providing a preliminary indication US11 may stabilize dsRNA during infection. However, no such increase was evident in HeLa cells (Fig 1D), perhaps because any effects are masked by the much higher levels of endogenous dsRNA present in these cells.

We found that vhs markedly reduced the protective effect of US11 on dsRNA without altering US11 protein levels, suggesting that it is able to destabilize US11-dsRNA complexes (Fig 5) in the same fashion that it destabilizes dsRNA in the absence of US11 (Figs 5 and 6). Vhs and US11 both accumulate to high levels after the onset of viral DNA replication, a period during which vhs exerts much of its inhibitory effect on PKR activation [14]. It therefore seems plausible that vhs-induced destabilization of US11-dsRNA complexes contributes to the overall reduction of dsRNA levels and PKR suppression mediated by vhs in HSV-infected cells. Further studies are required to directly test this supposition. Whether the RNA destabilizing proteins of other viruses similarly dampen an otherwise stabilizing effect of their cognate dsRNA binding protein is a basic and important question that awaits further investigation.

Vhs has been extensively characterized as an mRNA-specific RNase, and so its ability to destabilize dsRNA and dsRNA/US11 complexes was unexpected and raises many questions about the mechanisms involved. One possibility is that vhs accelerates the cellular pathways that normally recognize, process and/or degrade dsRNA, for example by recruiting the relevant host machinery to RNA duplexes via protein-protein interaction. However, it is difficult to envision how such a mechanism could overcome the protective effect of US11 on dsRNA. A second possibility is that vhs initiates the decay of annealed partially complementary mRNAs through normal vhs-dependent mRNA decay, followed by processing of the duplex regions by cellular factors. Here, we note that vhs is thought to initiate decay of mRNAs at least in part by making a limited number of cuts in the 5’ region [23, 25, 71, 72], followed by destruction of the mRNA body by the cellular 5’ -> 3’ exoribonuclease Xrn1 [18]. Quite remarkably, evidence suggests that Xrn1 may also play a role in destabilizing dsRNA, as indicated by the finding that Xrn1 knock-down potentiates the effects of the dsRNA mimic poly (I:C) [43]. A third possibility is that vhs might play a more direct role in destroying the dsRNA region, by “nibbling” in from one or both ends of the duplex, perhaps with the assistance of an RNA helicase. Here, it is very intriguing that vhs and the vhs orthologues of other α-herpesviruses display two conserved regions of extended sequence similarity to cellular structure-specific DNases, including XPG and flap endonuclease I (FEN-1) [73]. Perhaps vhs is able to cleave short regions of single-stranded RNA displaced from the end of a duplex RNA region, in a fashion analogous to the activity of FEN-1 on 5’ flap structures to remove the primers from Okazaki fragments during DNA replication [74].

These considerations suggest that defining the roles of vhs RNase activity and the known vhs co-factors eIF4A, eIF4H and Xrn1 in dsRNA decay will be highly informative. The eIF4A inhibitor hippuristanol inhibits the activity (Fig 6), suggesting that eIF4A is required. Key questions include whether eIF4A and its eIF4H co-factor function solely to target vhs to the capped 5’ ends of the partially complementary transcripts (as suggested in the second model) or instead actively unwind the duplex region (as in the third model), and whether Xrn1 and/or other cellular exoribonucleases contribute to its destruction. Determining the substrate requirements for the reaction will also be key. The partially complementary transcripts that we analyzed in our transfection assay are capped and polyadenylated, and are predicted to produce duplexes bearing 5’ and 3’ single-stranded extensions. It will be important to determine the roles, if any, of the cap and/or poly(A) tail, and whether single-stranded extension are required to initiate the decay of the dsRNA region. This information will clarify if vhs can promote decay of regions of dsRNA formed by annealing of partially complementary diverging (eg. UL23/UL24) and converging transcripts (eg., UL30/UL31), and if fully duplexed RNAs are also susceptible to attack.

Finally, our finding that vhs can destabilize dsRNA raises the possibility that the host-shutoff mRNA destabilizing proteins of other virus may have similar effects. Many plant viruses produce RNase III homologues to suppress RNA interference [75] and Lassa virus encodes a dsRNA-specific exonuclease [76], but the ability of viral mRNA destabilizing proteins to destabilize dsRNA during infection has not been examined. The VACV decapping enzymes act in conjunction with host Xrn1 to limit accumulation of dsRNA [43, 44], and it will be important to determine if this is achieved in part by destabilizing dsRNA.

## Material and methods

### Cells

HeLa cells (originally obtained from F.L Graham) and HFF cells (telomerase-immortalized human foreskin fibroblasts, a gift from Wade Bresnahan) were maintained in Dulbecco's modified Eagle Medium supplemented with 10% heat-inactivated fetal bovine serum (FBS), 100U/ml penicillin, and 100μg/ml streptomycin. Vero cells (ATCC) were grown in the same medium containing 5% FBS. Medium for Cre-Vero cells [77], stably expressing the Cre recombinase, contained additional hygromycin B (400μg/ml) to maintain the transgene.

### Viruses and BAC recombineering

We used the HSV-1 wild-type strain KOS (WT) and its vhs-deficient derivative ΔSma (Vhs^-^), which has 588-nucleotide deletion in the UL41 ORF [52]. We also used HSV-2 wild-type strain 333 (WT) and its vhs-deficient derivative vhsB (Vhs^-^[78]). The recombinant wild-type HSV-1 strain KOS37 was derived from the KOS37 bacterial artificial chromosome (BAC) containing the HSV-1 KOS genome ([77], obtained from David Leib). The KOS37 derivative containing a 588-nucleotide deletion in the UL41 ORF, KOS37ΔSma, (Vhs^-^) and two viruses expressing the true-late gene US11 from the immediate-early ICP47 (US12) promoter in a vhs-competent and vhs-deficient KOS37 background (IEUS11 and IEUS11Vhs^-^) have been described earlier [40]. The recombinant viruses US11^-^ and Vhs^-^US11^-^ designed for this study were created using *en passant* mutagenesis [79] of the KOS37 BAC to replace the 3^rd^ and 5^th^ codon of the US11 ORF with stop codons using the oligo pair 5’-TAAACATCTGGGTCGCCCGGCCCAACTGGGGCCGGGGGTTAGGTCTAGCTCATCTCGAGAGACACGGTAGGGATAACAGGGTAATGCATTT-3’ and 5’-GGGCGGAGGGTGGTTCCCCCCCGTGTCTCTCGAGATGAGCTAGACCTAACCCCCGGCCCCAGTTGGGGCCAGTGTTACAACCAATTAACC). A dual kanamycin/streptomycin selection marker [80] was used to introduce the 588nt deletion in the UL41 ORF of the US11^-^ BAC as previously described for Vhs^-^(ΔSma) [40]. The new BACs were transfected into Cre-Vero cells to generate infectious viral particles. Viruses were then passaged on Cre-Vero cells to remove the BAC sequence from the viral genome. All viruses were propagated and titred on Vero cells. Virus infections on HFF and HeLa cells were carried out at 37°C at a multiplicity of infection (MOI) of 10 PFU/cell.

### Plasmids

pcDNA3.1(+)mCherry was created by subcloning the mRFP derivative mCherry from pRSETmCherry (a gift from Roger Y. Tsien) into pcDNA3.1(+) (Invitrogen). pmCherry-SRPRα-forward (fwd) and pmCherry-SRPRα-reverse (rev) were created by inserting a 670bp EcoRV/StuI fragment of pSRPRα19N [71] into EcoRV digested pcDNA3.1(+)mCherry. This insertion is downstream of the mCherry ORF stop codon. Forward and reverse insertions of the SRPRα fragment were identified by restriction digest and confirmed by Sanger sequencing. pEGFP-US11 was created by inserting the US11 ORF into pEGFP-C3 (Clonetech). The US11 ORF was PCR amplified from KOS37 viral DNA with oligos 5’-CCCAAGCTTATGAGCCAGACCCAACC-3’ / 5’-AAACTGCAGCTATACAGACCCGCGAGC-3’

### Western blot analysis

Cell extracts were prepared and analyzed as previously described [36] using primary antibodies specific for the HSV-1 proteins gC (mouse; P1104; Virusys Corporation), gB (mouse; P1123; Virusys Corporation), ICP4 (mouse, P1101; Virusys Corporation), ICP34.5 (rabbit; gift from Ian Mohr), US11 (mouse; gift from Richard Roller), vhs (rabbit, AE328; gift from Duncan Wilson), UL47 (rabbit; gift from Gillian Elliott), thymidine kinase (goat, sc-28038, Santa Cruz), and the cellular proteins β-actin (mouse; A5441; Sigma), phospho-PKR (threonine 451; rabbit; ab81303; Abcam), PKR (rabbit; #12297; Cell Signaling), phospho-eIF2α (serine 51; rabbit; 9721; Cell Signaling), eIF2α (rabbit; 9722; Cell Signaling). Primary antibodies were detected using suitable secondary antibodies coupled to Alexa Fluor 680 (Invitrogen) or IRDye800 (Rockland) using the Odyssey infrared imaging system (LICOR) and ImageStudio Ver5.2 software.

### DsRNA dot blot

Our procedure was modelled on previous reports [81–83]. HFF and HeLa cells were mock infected or infected with the indicated viruses at MOI 10 for 12 hours. Total RNA was extracted using TRIzol reagent (Life Technologies) according to the manufacturer’s instructions. 1μg in 1μl was spotted on a Hybond^TM^-N^+^ membrane (GE Healthcare) and UV cross-linked using a Stratalinker 2400 (Stratagene). After blocking in 5% skim milk in TBS for 1 hour the membrane was incubated with the dsRNA-specific antibody (mouse, J2, Scions) in Odyssey blocking buffer (LICOR) at 4°C overnight. After several washes the membrane was incubated with a goat anti-mouse antibody coupled to Alexa Fluor 680 (Invitrogen) in 5% skim milk in Odyssey blocking buffer for 1 hour at room temperature. The dsRNA signal was visualized and quantitated using the Odyssey infrared imaging system (LICOR) and ImageStudio Ver5.2 software.

### Reporter assay for dsRNA

HeLa cells grown in 60mm dishes were transfected with 3μg pmCherry-SRPRα-forward (fwd) and 3μg pmCherry-SRPRα-reverse (rev) and 2μg of either pEGFP-C3 or pEGFP-US11 and Lipofectamine 2000 (Invitrogen) according to the manufacturer’s instructions. At 18–20 hours post transfection medium was replaced with fresh DMEM with 10% FBS for 2 hours. In some experiments, the cells were then treated with actinomycin D (Sigma, final conc. 2.5μg/ml) for 0, 2 or 4 hours or pretreated with actinomycin D for 30 min and infected at MOI 20 for 4 hours under continuous actinomycin D treatment.

### Digestion of RNA with single or double strand- specific RNases

RNA was extracted using 1ml TRIzol reagent (Life Technologies) according to the manufacturer’s instructions, then treated with DNaseI (Invitrogen/Ambion) for 15 minutes at 37°C. Samples were then incubated with a mix of RNase A/T1 (Invitrogen/Ambion) under high salt conditions (350nM NaCl_2_, 10mM TrisHCl pH7.5, 5mM EDTA) for 15 minutes at 37°C or RNase III (Invitrogen/Ambion) followed by RNase A/T1 (high salt) for 15min each at 37°C. RNA was extracted again using Trizol and 10 μg yeast tRNA as carrier. RNA digests on immunofluorescence samples were performed after the fixation and permeabilization step with RNase III/RNase III buffer or RNase A/T1 and high salt buffer for 15 min in the 37°C incubator.

### Northern blot

Digested RNA samples in H_2_O were denatured at 95°C for 5 minutes and mixed with ice-cold loading buffer. Total RNA samples from viral infections (10μg) were denatured in loading buffer at 65°C for 10 minutes. Samples were then subjected to 1.4% agarose-formaldehyde gel electrophoresis and transferred to a Genescreen membrane (NEN) and UV-crosslinked. Hybridization of the probe radio-labelled with ^32^P by random priming, was performed using ExpressHyb (Clontech) according to the user's manual. Signals were analysed with the Fujifilm FLA-5100 phosphoimager and Image Gauge Version 4.22.

Probes used: SRPRα probe (670bp EcoRV/StuI fragment of pSRPRα19N); probes for viral transcripts were PCR amplified from KOS37 viral DNA with the following oligo pairs: UL23 (5’-TTATACAGGTCGCCGTTGGG-3’ / 5’-GCGATACCTTATGGGCAGCA-3’), UL24 (5’-TCCAGAGCCTGTCCACGTAT-3’ / 5’-AAGCGGTGGTTAGGGTTTGT-3’), UL23/24 (5’-CAGCACCTGCCAGTAAGTCA-3’ / 5’-AGATCTTGGTGGCGTGAAAC-3’), UL30/31 (5’-CGTCGAATGTTGCATAGAGC-3’ / 5’-TTTACCCGGACCCCAATTAC-3’);

### Immunofluorescence microscopy

Transfected and infected cells grown on glass coverslips were fixed with 4% formaldehyde for 15 minutes at room temperature, permeabilized with ice-cold methanol for 8 minutes and blocked in 5% FBS in PBS. The primary antibody used was specific for dsRNA (mouse, J2, Scions, 1:50) and the secondary antibody was specific for mouse coupled to Alexa647 (Invitrogen). Cells were post-fixed with 1% formaldehyde for 5 minutes and mounted using Vectashield (Vector Laboratories Inc) supplemented with 1μg/mL 4′,6′-diamidino-2-phenylindole (DAPI). Digital images were acquired sequentially using a spinning disk confocal microscope (Wave FX, Quorum Technologies, Guelph, ON) integrated on an Olympus IX-81 inverted microscope base (Olympus, Richmond Hill, ON). Images were captured on an EM-CCD (ImageEM X2, Hamamatsu Photonics, Hamamatsu City, Japan) through standard fluorescence filter sets (Semrock, Inc., Rochester, NY). Representative images were prepared using Fuji software.

## Supporting information

S1 Fig DsRNA detected by J2 dotblot is sensitive to RNaseIII but not RNaseA/T1 digest. Total RNA of HeLa cells that were mock infected or infected with HSV-1 (KOS) WT and Vhs^-^ at MOI 10 was extracted at 12 hours post infection. RNA (1μg/1μl) was left untreated or digested with RNaseIII or RNaseA/T1 for 15min at 37°C, spotted onto the membrane, UV-crosslinked and processed like a Western blot. DsRNA was detected by staining with the dsRNA-specific antibody J2.
(TIF)

S2 Fig Cells transfected with the rev construct do not display increased levels of PKR activation. The figure displays the same gel as is shown in Fig 5D, expanded to show the data obtained with cells transfected with the rev construct. See Fig 5 legend.
(TIF)

S3 Fig US11 prevents detection of dsRNA by immuno-fluorescence with dsRNA specific antibody. HeLa cells were transfected with fwd/rev and pEGFP or pEGFP-US11, respectively. At 20 hours post transfection cells were analyzed by immunofluorescence with the dsRNA-specific antibody J2 (white).
(TIF)
