# Supplementary figures and images for: The herpes simplex virus host shutoff (vhs) RNase limits accumulation of double stranded RNA in infected cells: Evidence for accelerated decay of duplex RNA
Source: PLoS Pathog. 2019 Oct 18;15(10):e1008111. doi: 10.1371/journal.ppat.1008111 (PMC6821131; doi:10.1371/journal.ppat.1008111)

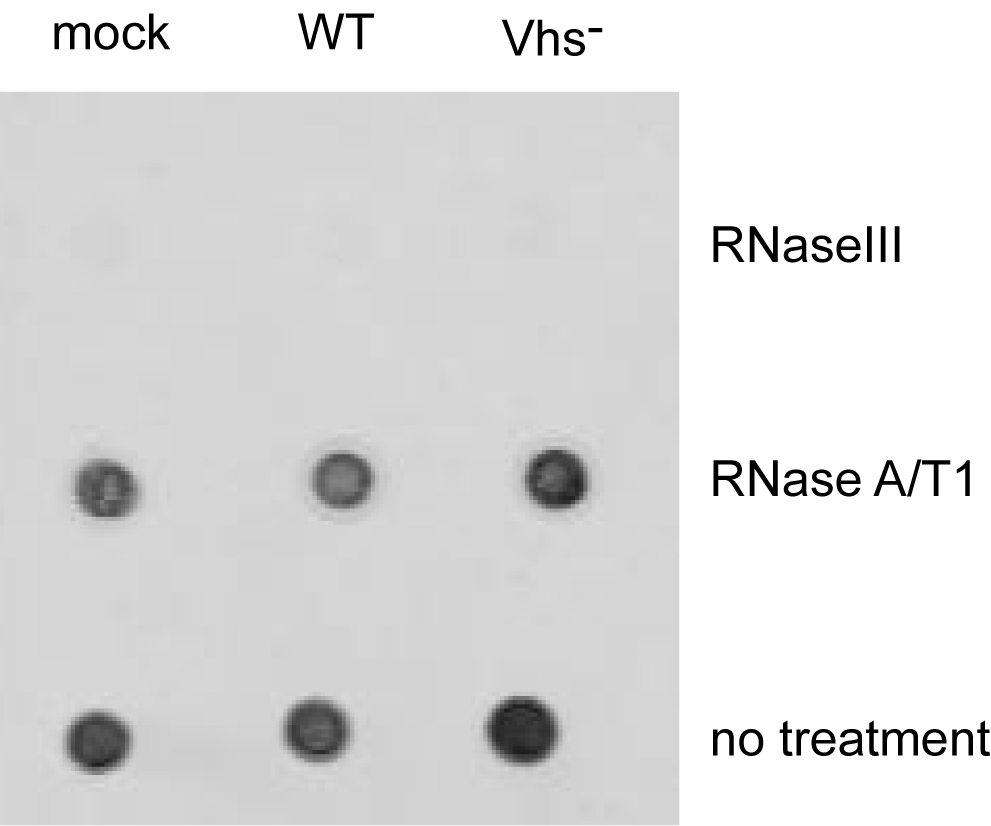

Supplement: S1 Fig — Total RNA of HeLa cells that were mock infected or infected with HSV-1 (KOS) WT and Vhs- at MOI 10 was extracted at 12 hours post infection. RNA (1μg/1μl) was left untreated or digested with RNaseIII or RNaseA/T1 for 15min at 37°C, spotted onto the membrane, UV-crosslinked and processed like a Western blot. DsRNA was detected by staining with the dsRNA-specific antibody J2. (TIF) [file ppat.1008111.s001.tif]

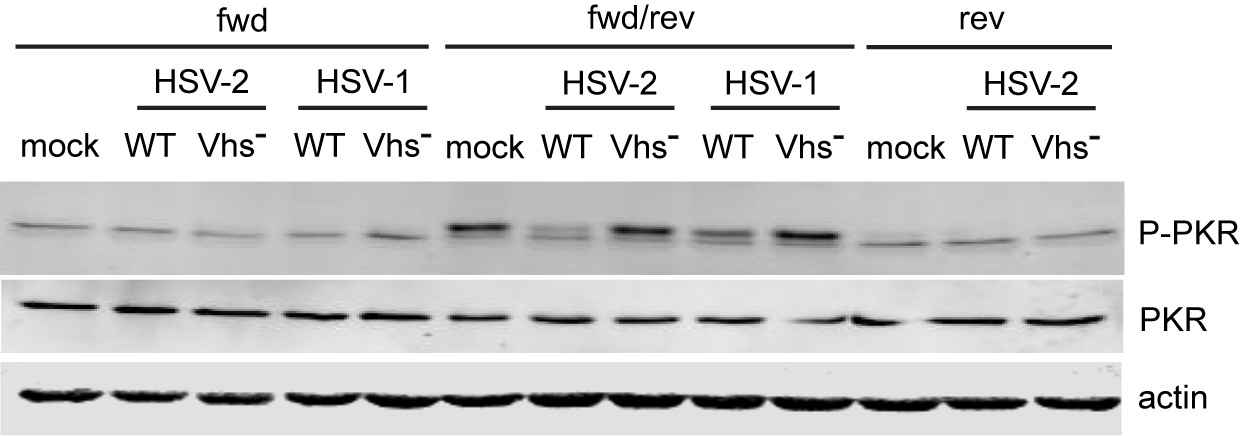

Supplement: S2 Fig — The figure displays the same gel as is shown in Fig 5D, expanded to show the data obtained with cells transfected with the rev construct. See Fig 5 legend. (TIF) [file ppat.1008111.s002.tif]

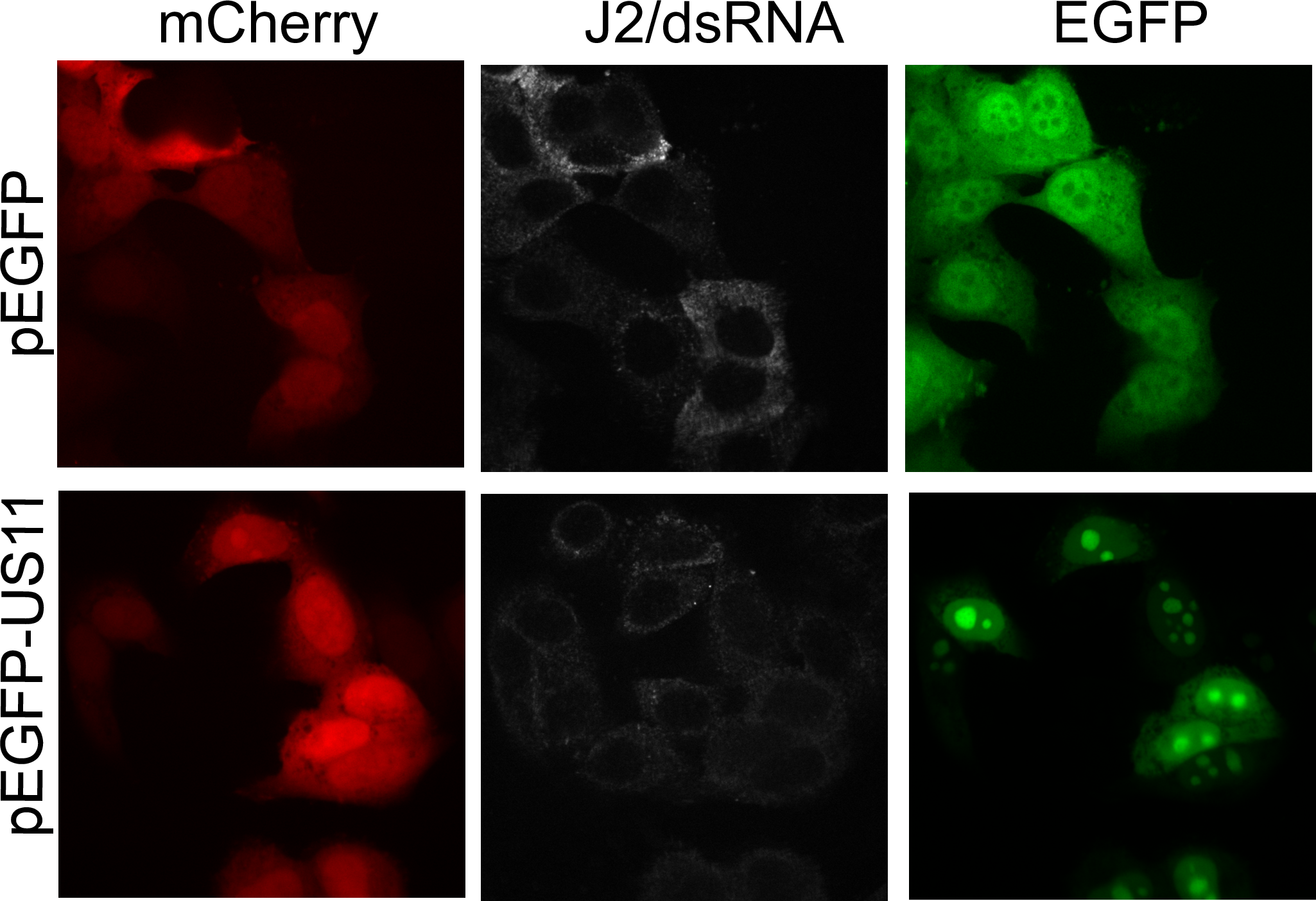

Supplement: S3 Fig — HeLa cells were transfected with fwd/rev and pEGFP or pEGFP-US11, respectively. At 20 hours post transfection cells were analyzed by immunofluorescence with the dsRNA-specific antibody J2 (white). (TIF) [file ppat.1008111.s003.tif]
